# Therapeutic play as a qualitative data generation method: A critical reflection

**DOI:** 10.4102/hsag.v24i0.1136

**Published:** 2019-07-11

**Authors:** Yolanda Havenga

**Affiliations:** 1 Adelaide Tambo School of Nursing Science, Tshwane University of Technology, Pretoria, South Africa

**Keywords:** Gestalt, HIV, lifeworld, Oaklander, qualitative research, therapeutic play, young children

## Abstract

**Background:**

Child-centred research requires researchers to develop research designs that will enable children to express their views in an ethical, appropriate and child-friendly manner and generate rigorous findings. These requirements challenge researchers to develop new methods to generate data with children, and the ‘younger the child, the more intense the challenge’.

**Aim:**

The aim of this article was to describe and reflect on the use of therapeutic play as a data generation method by nurse researchers with young children in a child-centred qualitative, explorative, descriptive and contextual study using a multiple case study strategy.

**Setting:**

The study was conducted with young children attending an outpatient department at a public hospital in Gauteng.

**Methods:**

Four young children, purposively sampled, participated in six sessions, each based on therapeutic play grounded in the work of Oaklander’s Gestalt play therapeutic approach, facilitated by a nurse researcher. Critical reflection was made about the play therapeutic approach as a data generation method, based on its ability to generate useful data, its implications for data analysis and its ability to be child-enabling and child-centred.

**Results:**

The play therapeutic approach, as a data generation method, is capable of generating useful data and amplifying children’s voices in the process.

**Conclusions:**

The nurse researcher needs to be highly skilled in this approach as it requires the ability to implement the specific play therapeutic approach in a safe and skilful manner.

## Introduction

Globally, child-centred research has gained momentum as researchers become increasingly aware of the importance of children’s perspectives on matters pertaining to them (Green 2012). This inclusion requires researchers to develop research designs that will enable children to express their views in an ethical, appropriate and child-friendly manner and generate rigorous findings able to inform practice (Kirk 2007). These requirements challenge researchers to develop new methods to generate data with children, and the ‘younger the child, the more intense the challenge’ (Dell Clark 2010:14).

Children’s voices should be central to research and to interpretations of research findings. There is a growing paradigm, which acknowledges that children could provide valuable information in research and that their perspectives should be included (Dell Clark 2010; Kirk 2007; Twycoss 2009) if child-friendly and appropriate methods are used (Einarsdóttir 2007).

The challenge for the researcher is, however, to find ethical research methods that would enable children to participate and be heard on matters pertaining to them (United Nations 1989). Such a child-centred approach requires research designs and data-generation methods to be individualised and tailored to children’s specific emotional, cognitive and social levels of development (Dell Clark 2010; Graue & Walsh 1998; Green 2012), respecting their dignity and opinions, being enjoyable (Bessell 2009; Green 2012), being culturally appropriate (Dell Clark 2010), and providing children with choices (Einarsdóttir 2007).

To tailor the approach, various existing and new methods of data-generation should be explored and evaluated. During the past 30 years various child-enabling methods have been described, such as activities of gesture, movement, play, body mapping, art techniques, photography, video recordings, sentence completion, diary completion and story writing (Dell Clark 2010; Kirk 2007; Punch 2002), book discussions, informal interviews, and puppets (Green 2012). The existing methods of generating data in research with children have not been considered sufficiently (Punch 2002). Reflecting on the current methods would enable researchers to refine and develop them and as Kirk (2007) points out could narrow the cultural gap between the adult researcher and the child participant. The advantages and disadvantages of these different methods, their usefulness and their implications for data analysis should be part of the reflexive process of all child-centred qualitative research (Punch 2002). In addition, when original methods for research involving children are considered, Kirk (2007) proposes that the researcher should evaluate the method based on its ability to encourage expression by the child, its ability to sustain interest and to correct the power imbalance between the adult researcher and child participant.

The aim of this article was to reflect on the use of one such method of data generation with young children, namely therapeutic play based on Oaklander’s Gestalt play therapeutic approach (Carroll & Oaklander 1997; Oaklander 1997, 2001, 2007), which was implemented by a nurse researcher in a study that aimed to explore the lifeworld of children living with HIV. To enable reflection, it is necessary to provide the backdrop for the reflection, namely the study, and explain the context wherein the method was applied.

## Background of the study

The need for this study was identified when, as an advanced psychiatric nurse practitioner, the researcher realised that a need existed to address the mental health requirements of young children living with HIV, based on an exploration of their lifeworlds. This need is important as health services provided to these children in South Africa are primarily designed for adults (Richter & Rama 2006). Adult-informed child health services could be based on assumptions that might be inappropriate for children (Australian Research Alliance for Children and Youth and New South Wales Commission for Children and Young People Commission for Children and Young People 2009). There was a need to generate knowledge, by working directly with young children who used these healthcare services to develop child-relevant mental health service delivery models (Christian et al. 2010; Dell Clark 2010; Kirk 2010).

The aim of the study was to describe the lifeworlds of young children living with HIV by means of therapeutic play. This information would then be used to develop a model for the facilitation of enhancing the mental health of young children living with HIV.

A child-centred qualitative, explorative and descriptive research design was used (Dell Clark 2010) based on a multiple case study strategy. The multiple case study strategy is suitable for descriptive and explorative research of this nature, and when research is carried out in a holistic and in-depth manner (Yin 2017). This strategy is further useful when multiple types of research evidence could be used and triangulated (Yin 2017) as in this case interviews, field notes and artefacts created by children were used. Four young children were sampled using homogeneous purposive sampling (Polit & Beck 2017), who were aged 3–7 years, HIV positive, on anti-retroviral treatment and accessing health services at a specific paediatric outpatient department in South Africa. Young children who were acutely ill during the time of data collection were excluded for ethical reasons. Four young children were selected to provide a rich description of the phenomenon based on what Yin (2017) describes as replication logic.

An appropriate and interactive data generation method had to be developed for young children finding it difficult to respond to direct questions. Such a method had to enable the nurse researcher to engage with the young children in a manner with which they were more familiar and comfortable (Aldiss et al. 2009). Children have a natural affinity for play and it is a natural form of expression of their feelings and thoughts (Axline 1974). Therefore, using therapeutic play as interviews was considered a suitable option. The concept interview is broad, involving one or more sessions of interaction between a researcher and interviewee that lasts between 30 minutes and 3 hours, is recorded, transcribed and subsequently analysed (Bedoin & Scelles 2015). Dell Clark (2010) suggests that age-appropriate ways of interviewing include the methods used by play therapists, such as props, visual artefacts, puppets, scale models and pictures. According to Dell Clark (2010), many of these methods have earned respect as data generation tools. Therapeutic play, based on Oaklander’s Gestalt play therapeutic approach (Carroll & Oaklander 1997; Oaklander 1997, 2001, 2007), hereafter also referred to as the play therapeutic approach, was implemented by a nurse researcher who was also an advanced psychiatric nurse practitioner trained in using this method. From a research perspective, this play therapeutic approach to data generation could be seen as a type of ‘unstructured interview’ (Dell Clark 2010) due to its existential, phenomenological and holistic nature (Carroll & Oaklander 1997; Oaklander 1997, 2001, 2007). Its phenomenological paradigm enables a focus on the present and on the child’s ‘perspective’ (Okun & Kantrowitz 2015). This approach has been described by Mortola (2006:xxix) as ‘a bridge for diverse professionals working with children and adolescents in varied cultural contexts’ and is suitable as an ‘unstructured interview’ based on its resemblance to qualitative research, its cross-cultural bearing and its open-ended nature (Mortola 2006).

In this play therapeutic approach, the phases followed in each session were based on seven steps according to Oaklander (1997, 2007): (1) building a therapeutic relationship, (2) contact-making, (3) building self-support in children, (4) emotional expression, (5) self-nurturing, (6) addressing the inappropriate process and (7) termination.

Both directive and non-directive play therapy methods can be integrated in this play therapeutic approach (Carroll 2009). However, for the purpose of this study, the greater focus was on the non-directive open-ended methods as a form of unstructured interviews, which promised to assist in the emergence of the young child’s lifeworld. A non-directive approach means that it is unstructured, thus the child’s responses are not prompted in any way (Jones, Casado & Robinson 2003).

A minimum of six therapeutic play sessions was conducted with each young child, which constituted the data for each case study. Oaklander’s Gestalt play therapeutic approach (1997, 2007) guided this process. The phases used for the purpose of data generation included relationship building, contact-making, creating opportunities for self-expression and termination. During these phases, the young children were engaged in different activities, according to each child’s needs and preferences at that time, for example body mapping, drawing and creating play dough figures. These activities emerged based on each young child’s preferences throughout the phases described and were not predetermined or limited to any one phase of the therapeutic sessions (Havenga 2011).

South Africa has 11 official languages. Most of the young children were Setswana speaking but the researcher was unable to converse in Setswana (although she could understand some of the communication), requiring the presence of a translator. The sessions were audio-visually recorded and held in a specifically prepared room in a paediatric outpatient department. Toys, art media and appropriate seating were available, and field notes were kept throughout the data gathering phase.

In each session, the following four phases ensued, although not in a linear and chronological manner, as both the nurse researcher and child moved back and forth according to the child’s needs and preferences (Oaklander 2007).

### Phase 1: Therapeutic relationship building

At the beginning of each of the six sessions, sufficient time was spent building a trusting relationship with the child by communicating in a friendly and playful manner, sitting with the child on the floor and explaining the structure of the sessions and the roles of everybody present. Much of the first session was devoted to the building of a trusting relationship, which Oaklander (1997) explains lays the foundation for the rest of the therapeutic play sessions. In this study, a relationship also had to be built between the child and the translator. Additionally, a relationship was established with the young child’s parent who was available during the play session but in a separate room from the child (Havenga 2011).

### Phase 2: Contact building

Establishing contact with children during the therapeutic process is a precursor for expression of the self (Oaklander 1997) – the phase in which the researcher intended to get the most information about the young child’s lifeworld. Contact in this context implies working with the child’s resistance, facilitating sensory awareness and making contact with his or her own body (Oaklander 1997).

The young children engaged in activities where their senses were stimulated, body movements explored and feelings discovered. Examples of sensory activities included finger painting, playing with water and sand, smelling flowers, touching different textures and making music. Some of the body activities included playing with a ball, doing exercises in front of a mirror and blowing soap bubbles. Resistance in the relationship (Oaklander 2007) was respected by accepting children’s preferences or requests to terminate activities and if a child chose not to respond to a question the nurse researcher respected this and did not pursue this line of questioning or conversation any further.

### Phase 3: Creating opportunities for self-expression

Opportunities were created for the young children to express themselves through creative, expressive, projective and dramatised play (Oaklander 2001). It is in this phase where most data were generated. This play was self-directed and the researcher carefully assessed whether she was ‘allowed’ to join in by asking each child’s permission to participate and by observing his or her acceptance of her playing along through their non-verbal communication. During this time, clarifying questions explored the meaning of games and pictures. Long periods of silence were also present when a young child could play freely (Havenga 2011).

Creative expressive techniques were used to create opportunities for self-expression such as painting or drawing pictures, making sand tray scenes or making objects from play dough. These projections of symbolic material allowed expression of the young child’s self (Oaklander 1997) and drawing and painting served as a basis for communication with the young child (Bach in Bertoria 1993). The focus was not on the young children’s artistic abilities, but on the meaning-making process related to these expressive activities (Green 2012).

The children remained the analysts of their created artefacts. The role of the nurse researcher was to guide the children carefully to a verification of their understanding of the drawing or other artefacts created (Oaklander 2007) and use it as a communication starting point. As Mitchell (2006:63) cautions, drawings should be a ‘departure point for apprehending something of their [children’s] worlds and world-making’. Bertoria (1993) and Oaklander (2007) caution therapists, and in this case researchers, to be tentative about children’s drawings as they might read something of themselves into a picture.

### Phase 4: Ending the therapeutic play sessions

To make closure of the session evident to the children, they were asked to assist in tidying up the playroom as a termination activity that, Oaklander (1997) suggests, should be used to enable children to pre-empt the ending of the session. Children were closely observed for distress on termination and, in some cases, sessions continued with a more gradual process of termination (Havenga 2011).

After all sessions were transcribed and observation field notes were added, each case study was *analysed* separately whereafter a cross-case analysis (Yin 2017) was performed. *Trustworthiness* was enhanced through the criteria of credibility, transferability, dependability, confirmability and authenticity (Lincoln & Guba 1985).

The findings of the study revealed that the lifeworld of the young children living with HIV centred around needs developing and being met in relationship with themselves, others and the environment. Making contact in these relationships was important in meeting their own needs. Pathways to contact were affected by their relationships with themselves, others and factors within their environment (Havenga 2011).

### Ethical considerations

*Ethical* approval was obtained from the appropriate international review board, provincial authority, hospital and clinic manager. Ethical approval was granted by University of Johannesburg ethics committee (reference number: 76/07). Consent from parents and assent from children were obtained at the beginning but was a continuous process before and during the sessions as Gibson and Twycross (2007) propose it not be a once off event. To ensure confidentiality, names and identifiable information were removed from all the reports and the translator signed a confidentiality agreement. As case studies have the potential to violate anonymity, slight changes were effected to biographical information without influencing the context of the study.

Protection from harm was ensured by including parents in the sessions if they wished to attend, or if the child requested their parents’ presence (Havenga 2011). When working with young children the inclusion of parents can be beneficial to establish a comfortable environment and to support children’s need for protection (Green 2012). The environment was child-friendly and the researcher was skilled in implementing the specific therapeutic play approach. If a child was ill the session was postponed, which happened only once during the data collection period.

### Measures to ensure trustworthiness

To enhance trustworthiness the criteria credibility, dependability, confirmability, transferability and authenticity were adhered to by means of:

- Prolonged engagement and persistent observation of children.
- Peer debriefing.
- Use of an independent coder.
- Dense descriptions of the findings and methodology.
- Keeping an audit trail (Polit & Beck, 2017).

## Reflection on therapeutic play as a data generation method

Following the description of the study and the specific play therapeutic approach as a data generation method, a critical reflection of this method will be carried out based on the following criteria formulated as questions (Dell Clark 2010; Kirk 2007; Punch 2002):

- Does the method generate useful data?
- What are the implications of the method for data analysis?
- Is the method child-enabling and child-centred by:
  ■ encouraging expression by the children?
  ■ sustaining children’s interest?
  ■ addressing the power imbalance between the adult researcher and the child participant?

### Does the method generate useful data?

This method’s usefulness was evaluated against its ability to generate data, which sufficiently represented each child’s perspective, answered the research question and provided data that were trustworthy.

The research question was ‘What is the life-world of the young child living with HIV?’ The findings can be summarised in the following excerpt:

The life-world of young children living with HIV unfolded as a relational life-world of integrated, holistic, dynamic and interactive relationships with themselves, others and the environment. Within these relationships, the need to feel capable, autonomous, independent and safe, both arose and were met. The young children’s ability to meet their own needs in a developmentally appropriate manner hinged on their ability to make contact in these relationships. The pathways enabling contact were influenced by various intrapersonal, interpersonal and environmental factors. (Havenga 2011:234)

From the summarised findings, one can deduce that the concept of HIV did not come to the fore clearly, but that the data related to a more general lifeworld discussion and included a central concept to the Gestalt Theory, namely contact (Yontef & Jacobs 2008), the theoretical framework from which the researcher was departing. The reasons for HIV not emerging in the data could be many. Firstly, the non-directive approach followed implies the nurse researcher did not follow a specific agenda, predetermined plan or a schedule of games and questions focussed on eliciting information specifically about HIV in the child’s lifeworld. The nurse researcher did, however, gently probe during play, through questions related to medical visits, body mapping activities and toys that represented the medical context. Some children used the stethoscope to listen to their own, the dolls’ and the researcher’s heart and some administered oral medication to the dolls. Green, Crenshaw and Lubin Langtiw (2009) describe this as a form of healing and repair play. Punch (2002) states that a young child’s limited vocabulary could limit their understanding of the dialogue with the researcher, but the researcher could possibly not understand the child’s vocabulary – even more so if dialogue was through translation. Other possible reasons for HIV not emerging in the findings were that young children might not be fully able to conceptualise what HIV meant (Wiener, Havens & Yiu Kee Ng 2003) or that HIV did not play such an important role in the lifeworld of the young child.

The non-directive approach, despite its limitations to illuminate the phenomenon of HIV was beneficial as it limited the nurse researcher’s influence to focus on the phenomenon under study and enhanced participation by the child. Participatory methods must be designed and implemented in such a way as to allow children to take the lead (Sommer, Pramling Samuelsson & Hundeide 2010) and enable children to exercise some level of control within the research process (Green 2016). The multiple methods and phases of play enhanced rigour as combining methods (such as drawing and play) enhances method triangulation (Bessell 2009; Leonard 2006; Polit & Beck 2017). A further advantage of using multiple methods in a qualitative study of this nature is that the researcher and child could consider methods appropriate for a specific child at each session. Flexibility of the data collection method, therefore, required adaptations made by the researcher during the research process (Green 2012; Kirk 2007). Such adaptations also pertain to the concern that the unique methods used with children, might only serve to build relationships with children rather than to generate useful data. This potential consequence could be minimised in the play therapeutic approach (presented in this article) as data-generation method, as not only one tool was used to enhance self-expression by the children and as a number of sessions were conducted. Building lasting relationships with the child participants and their parents is, however, important as it promotes the ability to obtain informed consent or assent and assists in facilitating more personal and open expression by children when participating in research (Bedoin & Scelles 2015).

Was the research question addressed by the method? The method was able to elicit a lifeworld description of the young child (who happens to also live with HIV). However, if HIV (as main role player) in the lifeworld of the child is explored, this method was not effective as a more directive approach to play, asking about and specifically exploring HIV, might be more appropriate to do so.

### What are the implications of the method for data analysis?

Text from transcripts was analysed using descriptive content analysis (Polit & Beck 2017). Using transcripts was, however, challenging when there was limited content in the dialogue. Often there was more depth in the process that transpired during the sessions and the ways in which the young children played or participated in creative activities (Havenga 2011). Such data were evident in the field notes written by the nurse researcher, based on her observation and interpretations of the processes formulated within the play therapeutic paradigm. The analysis, therefore, depended on dense description of the field notes. In their work with interviewing children with communication disorders, Bedoin and Scelles (2015) explain that immersion with each interviewee is important and to achieve this immersion, the use of non-verbal and visual data is required in addition to the transcriptions in the analysis.

Within this theoretical framework, artefacts could not be interpreted, making them redundant during the data-analysis process, when they were not explained by the children during the data-collection phase. During the therapeutic sessions, the nurse researcher explored the meaning of artefacts such as paintings and play dough figurines but the young children rarely responded to these questions or owned any of the projections. This was possibly because of their young age, the translation process, their personal process (such as shyness) and the unfamiliarity with the therapeutic process. Despite the limitations of this method in data analysis, it is beneficial for preventing adult interpretations from dominating the descriptions of children’s experiences and it includes the child in the process of analysis (Bessell 2009).

### Is the method child-enabling and child-centred by encouraging expression by children?

This data generation method employed various phases and methods, which encouraged expression as children could identify the most suitable activity for their self-expression.

In phase three of the play therapeutic approach, opportunities were created for the expression of emotions. However, expression was not limited to this phase. When the child’s expression is inhibited, this approach affords the opportunity for moving back and forth to the phase of contact-making with him or her (Oaklander 2007). During the sessions, the nurse researcher noticed that the use of sensory activities and movement were particularly helpful for enhancing children’s abilities to express themselves. For example, when one child was frustrated at the beginning of a session, physical activities such as blowing soap bubbles and playing with a ball enabled him to later play more openly and express themes such as concerns about crime and safety in his neighbourhood.

The use of various non-verbal methods such as drawing, painting and model building in collaboration with discussion encourages expression by young children who have not fully developed their language ability, or where there is a language barrier as was the case between the children and nurse researcher in this study. Using visual methods is particularly useful to enable researchers to tap into the lived experiences and lifeworlds of children who are challenged, for example traumatised or with an intellectual disability (Literat 2013).

### Is the method child-enabling and child-centred by sustaining the children’s interests?

Children chose the object of play, the tempo and the content. The nurse researcher made various mediums for play available which the young children selected according to their personal interests, needs and circumstances. The flexibility of this approach and various tools for data generation enabled children to engage in activities that interested them. Dell Clark (2010) confirms that combining creative art activities with verbal tasks enhances children’s attention span and supports their interest and involvement (Dell Clark 2010).

When the young children lost interest in a specific activity or method, they had the freedom to ‘move on’ and choose another activity, which they found to be more stimulating or interesting. Some children preferred playing with water or sand whilst others preferred playing with a ball or blowing soap bubbles. Most children preferred playing with the human figures in the miniature dollhouse and often spent most of the session doing this specific activity. Activities children in this study did not seem to enjoy much were drawing or playing with the play dough. Punch (2002), Dell Clark (2010) and Einarsdóttir (2007) caution that not all children might enjoy drawing as they are not equally skilled, and they might associate drawing with the school environment where they are often evaluated (Dell Clark 2010). The multiple tools used for data-generation and phases in this play therapeutic approach are beneficial as they enable the children to choose what interests them and accommodates an individualised approach to data generation (Leonard 2006; Kirk 2007).

### Is the method child-enabling and child-centred correcting the power imbalance between the adult researcher and child participant?

In many societies, there is a power imbalance between adults and children, which might be even more pronounced in the healthcare provider-child relationship. Bedoin and Scelles (2015) explain that this asymmetry in the relationship between the child and the researcher is not only related to age but also to the status of the researcher. This power imbalance between the adult researcher and the child participant influences what data are collected and interpreted (Green 2016).

Dell Clark (2010:80) stresses the importance of correcting these power imbalances when doing research with children by stating: ‘One of the most important principles for the child-centred interviewer, then, is to break the usual norms in order to power share with the child.’ Means to break such power imbalances are research approaches that underline the importance of establishing trust with children, creating a comfortable environment and providing children with choices (Green 2016).

This play therapeutic approach emphasises the nurturing of the I/Thou relationship between the child and therapist (in this case the nurse researcher), which implies meeting on an equal level with the child. Maintaining equality is the nurse researcher’s responsibility and is enhanced through a non-judgemental attitude and following the child’s pace (Oaklander 2001, 2007). In this study, children were initially hesitant to interact with the researcher; however, later they became more open and engaging. This was possibly due to these internalised power imbalances between adults and children (Punch 2002) and not being used to adults asking for their opinions or playing with them (Einarsdóttir 2007). To potentially mitigate this effect, the nurse researcher did not wear a nurse’s uniform and sat with the children on the floor. She asked permission to play with the young child and never touched a toy without his or her permission nor told any child how or with what to play.

The nurse researcher noticed that the way in which the parents prepared their children for the play sessions affected the power imbalance. One parent told her child that the researcher was a teacher, which slowed down the process of developing an equal relationship as teachers are viewed as powerful, in control and able to evaluate and reprimand children (Dell Clark 2010).

## Conclusion

In this article, the researcher described and reflected on the use of therapeutic play as a data generation method used by a nurse researcher with young children in a child-centred qualitative, explorative, descriptive and contextual study in South Africa. The aim was to describe this method and reflect on its usefulness, the implications for data-analysis and whether it is child-enabling and child-centred.

The data generation method is useful for its ability to elicit the child’s perspective. This was the case because as the non-directive nature during the sessions and the limitation to interpretation during data analysis prevented the adult researcher’s perspective from overshadowing that of the child. Limiting the researcher’s influence further enhances the credibility, authenticity and confirmability of the findings. Furthermore, the use of multiple phases, methods and moving back and forth between these phases to generate data enhances credibility of the findings through method triangulation. The use of various methods, that are not all centred in dialogue, enables ‘dialogue’ between the young children and the nurse researcher across cultural and language barriers.

The child-centeredness of the method is enhanced by the flexibility of multiple methods, which could be used according to each child’s preferences, interests and level of development. This flexible method enables both the nurse researcher and the young child to choose methods and techniques that make them feel competent and which interests them (Einarsdóttir 2007). Such methods afford children a voice as great effort is taken to build and maintain equal relationships with the young children, enhancing trust and open expression.

Eliciting an authentic child perspective does, however, have limitations as the information is interpreted from an adult perspective through the theoretical perspective, in this case a Gestalt theoretical lens. It is, therefore, important that the nurse researcher explicitly positions herself in terms of the paradigm and theoretical framework wherein the research is conducted. The field notes are an important part of the generated data and are written by the nurse researcher as he or she describes the young child’s behaviour and process of each therapeutic session. The non-directive nature, however beneficial, might limit the researcher’s ability to obtain information about a specific theme, as in this case where HIV did not come to the fore in the current study’s findings.

Future researchers might want to learn more about the directive dimension of this therapeutic play approach and triangulate it with the non-directive dimension. Such an approach could help to obtain information about a specific topic as in this case focussing more on HIV in the young child’s lifeworld. This play therapeutic approach, as data generation method, should be carefully considered as the nurse researcher has to be competent in its safe use and should receive continuous supervision during the process. Also, parents require guidance when they prepare their young children for these sessions to ensure that the researcher’s identify in the field is not misconstrued.

Oaklander’s Gestalt play therapeutic approach (1997, 2007) as a new data generation method is child-centred and enabling. Its usefulness for generating data has both benefits and limitations, which researchers should carefully consider.

The versatile and flexible nature of the play therapeutic method, as a data generation tool, enables children through a combination of visual, body and verbal expressions to be a useful ‘expressive channel to voice their [young children’s] inner stories’ (Literat 2013:85).
